# Building global capacity for COVID-19 vaccination through interactive virtual learning

**DOI:** 10.1186/s12960-022-00710-7

**Published:** 2022-02-04

**Authors:** Shoshanna Goldin, Nancy Hood, Alexandre Pascutto, Celine Bennett, Ana Carolina Barbosa de Lima, Nicole Devereaux, Aleksandra Caric, Karan Rai, Shalini Desai, Ann Lindstrand, Bruce Struminger

**Affiliations:** 1 World Health Organization, Geneva, Switzerland; 2 Project ECHO, University of New Mexico, Albuquerque, USA

**Keywords:** Workforce training needs, COVID-19, Capacity building, Peer-to-peer learning, Digital learning

## Abstract

**Background:**

To support the introduction of the COVID-19 vaccine, the World Health Organization and its partners developed an interactive virtual learning initiative through which vaccination stakeholders could receive the latest guidance, ask questions, and share their experiences. This initiative, implemented between 9 February 2021 and 15 June 2021, included virtual engagement between technical experts and participants during a 15-session interactive webinar series as well as web and text-messaging discussions in English and French.

**Methods:**

This article uses a mixed-methods approach to analyze survey data collected following each webinar and a post-series survey conducted after the series had concluded. Participant data were tracked for each session, and feedback surveys were conducted after each session to gauge experience quality and content usability. Chi-square tests were used to compare results across professions (health workers, public health practitioners, and others).

**Results:**

The *COVID-19 Vaccination: Building Global Capacity* webinar series reached participants in 179 countries or 93% of the WHO Member States; 75% of participants were from low- and middle-income countries. More than 60% of participants reported using the resources provided during the sessions, and 47% reported sharing these resources with colleagues. More than 79% of participants stated that this initiative significantly improved their confidence in preparing for and rolling out COVID-19 vaccinations; an additional 20% stated that the initiative “somewhat” improved their confidence. In the post-series survey, 70% of participants reported that they will “definitely use” the knowledge derived from this learning series in their work; an additional 20% will “probably use” and 9% would “possibly use” this knowledge in their work.

**Conclusion:**

The *COVID-19 Vaccination: Building Global Capacity* learning initiative used a digital model of dynamic, interactive learning at scale. The initiative enhanced WHO’s ability to disseminate knowledge, provide normative guidance, and share best practices to COVID-19 vaccination stakeholders in real time. This approach allowed WHO to hear the information needs of stakeholders and respond by developing guidance, tools, and training to support COVID-19 vaccine introduction. WHO and its partners can learn from this capacity-building experience and apply best practices for digital interactive learning to other health programs moving forward.

## Background

Introducing the COVID-19 vaccines was the largest simultaneous vaccine deployment initiative in history. As such, the global immunization community needed mechanisms to access real-time normative guidance and implementation examples. Through the Access to COVID-19 Tools (ACT) Accelerator’s Country Readiness and Delivery (CRD) workstream, the World Health Organization (WHO) and partners developed resources to support countries in preparing for and implementing COVID-19 vaccination at scale [1, 2]. These resources included three self-paced massive open online courses (MOOCs) available through the OpenWHO platform and accompanying job aids to support health workers and immunization professionals in deploying and administering the vaccines.

While the MOOCs and job aids were well received by the global, national, subnational, and regional participants, they requested interactive training to support their preparation for COVID-19 vaccine deployment. In response, WHO collaborated with Project ECHO, TechNet-21, the United Nations Children’s Fund (UNICEF), the Sabin Institute’s Boost Community, and the COVID-19 Vaccine Equity Project to develop the *COVID-19 Vaccination: Building Global Capacity* virtual learning initiative [3, 4]. Implemented between 9 February 2021 and 15 June 2021*,* the aim was to reach a global audience, with a particular focus on supporting immunization focal points in low- and middle-income country (LMIC) settings.

The initiative leveraged Project ECHO’s nearly 20 years of experience using a virtual, case-based “community of practice” approach to promote real-time, peer-to-peer learning among health workers and public health practitioners. The Project ECHO learning model promotes clinical and public health collaborative learning using videoconferencing in an “all teach, all learn” approach on a regional, national, or international scale. This model has been used to improve care and outcomes for chronic and acute health conditions; it has also supported the COVID-19 and other emergency public health responses globally [5–13].

The *COVID-19 Vaccination: Building Global Capacity* initiative included virtual engagement between technical experts and participants using a multipoint videoconferencing platform, with real-time polling, chat, and Q&A functions, in addition to instant messaging group communication and discussion fora in English and French on TechNet-21, a virtual global network of immunization providers. The aim was to create a learning ecosystem of real-time and asynchronous peer-to-peer learning networks through which health workers, public health practitioners, and other COVID-19 vaccination stakeholders could receive the latest guidance, ask questions, and share their experiences with COVID-19 vaccine introduction.

A growing body of evidence supports the effectiveness of webinars in advancing the knowledge of participants, and providing a safe and supportive environment for learning [14]. Interactive engagement and knowledge gain through webinars promote student learning as effectively as in-person training [15]. For example, in a webinar series in the Republic of Korea, a majority of participants reported a higher likelihood of participating and asking questions in a virtual format compared to a live lecture [16]. Participants have also been reported to appreciate the direct, real-time communication with other participants and facilitators offered through a webinar platform [17].

The COVID-19 pandemic accelerated the already increasing use of webinars to transfer information and train participants. However, concerns have been raised about limited access to workplace-based learning experiences, and the effectiveness of technology-based competence assessments [18]. While physicians in one survey reported feeling overwhelmed by the number of virtual meetings, they also expressed appreciation for the increase in international conferences and virtual courses now available from home during the COVID-19 pandemic [19]. During the pandemic period, medical training seminars have experienced rapid growth in attendance as well as positive feedback and active participation from learners [20]. Medical didactic seminars have been reported by the participants to be “very beneficial,” with a majority (61%) of participants preferring the virtual format to in-person conferences [21].

This paper contributes to the literature on interactive digital learning by describing participation in and outcomes from the *COVID-19 Vaccination: Building Global Capacity* initiative. The mixed-methods approach and findings provide insight into how this webinar series supported COVID-19 vaccination capacity building, particularly in LMICs.

## Methods

### Initiative development

The *COVID-19 Vaccination: Building Global Capacity* learning series had two objectives: to amplify use of and receive feedback on the ACT Accelerator CRD workstream’s COVID-19 vaccine introduction guidance, tools, and training resources; and to share best practices and lessons learned on key aspects of COVID-19 vaccine introduction and administration through case studies and peer-to-peer engagement. The 15 one-hour learning sessions were divided into two subseries, complementing the resources developed by the ACT Accelerator’s CRD workstream led by WHO, UNICEF, and Gavi, the Vaccine Alliance (Gavi). Five of the sessions focused on topics relevant for health workers, while 10 of the sessions focused on topics relevant for national and subnational stakeholders. Participants were recruited through email announcements and program websites, hosted by TechNet-21 and Project ECHO, to join the sessions relevant to their professional or personal interests. Certificates were provided for each session attended. Participants were not expected or required to attend every session.

The live webinars supplemented the OpenWHO MOOCs launched in December 2020 for national and subnational stakeholders preparing for COVID-19 vaccine introduction and health workers responsible for safe administration of COVID-19 vaccine (Annex 1) [22, 23]. The webinars were complemented by TechNet-21’s web discussion fora and cloud-based instant messaging groups in English and French. These fora allowed peers and webinar organizers to interact between sessions. By the end of the webinar series, approximately 1000 members were participating in the English or French channels of the instant messaging mobile platform; these instant messaging discussion groups have actively continued beyond the end of the webinar series.

The overarching goal of this initiative was to make COVID-19 vaccination information transparent and readily accessible to all. As such, the sessions were marketed through mailing lists, social media, the TechNet-21 and Boost online platforms, and word of mouth. Registration for the sessions was open to the public and unrestricted. Session materials (i.e., recordings, presentations, and responses to questions posed during the session) were posted on publicly accessible websites, such as those hosted by TechNet-21 and Project ECHO, and shared on the mobile channels in English and French, to facilitate access.

Each session included presentations or panel discussions by technical experts and/or peer learning through sharing of country case studies as well as allocated time for questions from participants. The subject matter experts responded to participants’ questions in writing or verbally during the live webinars; selected questions submitted during the registration process were also addressed. Questions that were not addressed during the webinar due to lack of time were collected and sent to the presenters and subject matter experts for compilation into a Q&A document that was shared by email with all participants and posted on the TechNet-21 platform. Participants’ questions and recommendations via the post-session surveys, in addition to the ACT Accelerator’s identification of COVID-19 vaccination bottlenecks, were used to determine the topics that would be beneficial to focus on during the subsequent sessions.

### Participant demographics and attendance

Demographics were collected when participants registered for the webinars. Participants provided their name, email, organization, city, profession, and country or region. Attendance reports were automatically saved through the unique webinar registration link received by each participant. Webinar registration and attendance data were linked using the participant’s email address. Participants had the option to self-report their profession from a list provided.

### Surveys

A link to an electronic survey was shared with all webinar participants during and after each session. The post-session survey gave participants the opportunity to provide feedback about each learning session and receive an attendance certificate. The survey asked participants about their knowledge of the session’s topic before and after; relevance of the session to their current work; balance of lecture and interactivity; and intention to use what was learned in their work. In addition, Project ECHO distributed a final post-series survey link during the last session and in two subsequent emails to all program registrants to solicit feedback about the entire learning initiative, regardless of the number of sessions they completed. Survey questions are available in Annex 2. The University of New Mexico Health Sciences Institutional Review Board approved this evaluation (ID 20–469) and its consent process.

### Analyses

Chi-square tests were used to compare results across professions (health worker, public health practitioner, and other) and participating countries’ World Bank income status. The Wilcoxon signed-rank test was used to compare knowledge before and after sessions. Quantitative analyses were conducted in SPSS 28.0. Open-ended text responses were coded systematically using NVivo 1.4.1.

## Results

From 9 February 2021 until 15 June 2021, 3058 individuals from 179 countries participated in at least one webinar with similar numbers of health workers and public health professionals (Table 1); there were 6893 additional views of recordings of the webinars through YouTube. The *COVID-19 Vaccination: Building Global Capacity* webinar series reached participants in at least 93% of WHO Member States. The majority of the participants who joined the webinar sessions were from LMICs (Table 1).

**Table 1:** Webinar participation and country income by participant profession

	Health worker	Public health	Other	Total*
Participation				
Total	1469 (48.0%)	1336 (43.7%)	253 (8.3%)	3058
Attended 2 + webinar sessions	705 (48.0%)	572 (42.8%)	96 (37.9%)	1373 (44.9%)
Attended health-worker-focused sessions	1012 (52.7%)	699 (36.4%)	210 (10.9%)	1921
Attended national/subnational-focused sessions	954 (44.9%)	1029 (48.4%)	144 (6.8%)	2127
Country income				
Low-income	334 (48.1%)	325 (46.8%)	35 (5.0%)	694
Lower-middle income	545 (46.7%)	514 (44.0%)	108 (9.3%)	1167
Higher-middle income	215 (51.2%)	167 (39.8%)	38 (9.0%)	420
High-income	373 (48.6%)	322 (42.0%)	72 (9.4%)	767

The attendance size in the live sessions ranged widely depending on the topic; the most-attended session (932 participants) focused on interpersonal communications for COVID-19 vaccination, while the least well attended session (185 participants) focused on adaptive leadership for COVID-19 vaccine introduction. Since the participants attended an average of 2.2 sessions, the total number of attendances (> 6600) is substantially larger than the total number of unique individuals (3058) who participated in the live webinar sessions. Nearly 45% of participants attended more than one session, with the highest repeat attendance rates for health workers (Table 2). The response rate for post-session surveys was 46.4% and was 7.5% for the post-series survey. More than 76% of the post-series survey respondents attended four or more sessions compared to 14.7% of all participants.

**Table 2:** Webinar session experiences by participant profession

	*n*	Total	Health worker	Public health	Other	*P* value
Post-session surveys (n = 3067)						
Right balance of didactic and interactive learning	3023	2719 89.9%	1393 90.6%	946 89.2%	380 89.4%	0.437
Knowledge increased (before and after)	3021	1945 64.4%	996 64.8%	689 64.9%	260 61.5%	0.400
“Very” or “extremely” relevant to work	3026	2437 80.5%	1209 78.6%	910 85.6%	318 74.8%	< 0.001
“Definitely” plan to use what was learned	3023	2120 70.1%	1050 68.4%	806 75.8%	264 62.3%	< 0.001
Post-series survey (n = 228)						
Participated in all three WHO training formats	221	115 52.0%	54 50.0%	50 57.5%	11 42.3%	0.334
Met needs “a little” or “a lot” better than other COVID-19 trainings	225	167 74.2%	78 71.6%	70 77.8%	19 73.1%	0.602
Confidence increased “a lot”	225	179 79.6%	79 72.5%	76 86.4%	24 85.7%	0.038

*Some participants joined both series, so the overall participant total is lower than the sum of the two series

Participants used a variety of learning strategies. Of the 228 respondents who completed the post-series survey, 51.9% reported participating in all three of the COVID-19 vaccination training opportunities: the mobile based channels, OpenWHO COVID-19 MOOCs, and the *COVID-19 Vaccination: Building Global Capacity* webinars. Respondents from low-income countries were more likely to report participating in all three training types (68.0% vs. 43.3%, *P* < 0.001).

**Fig. 1:**
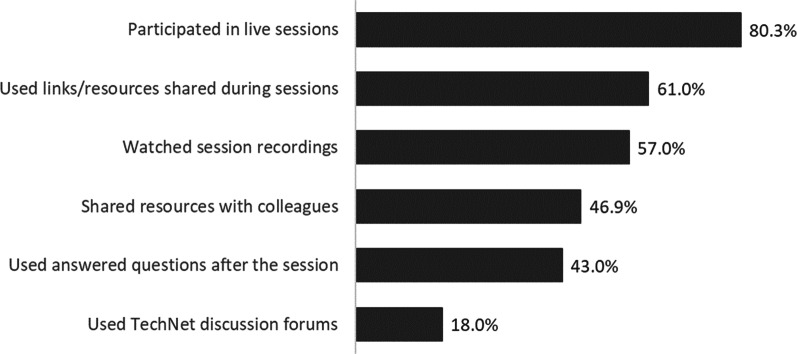
Resources used by participants (post-series survey respondents, *n* = 228)

Post-series survey respondents reported using information in various ways after the session, including watching session recordings and sharing resources with colleagues (Fig. 1). Of the 228 post-series survey respondents, 61 gave examples of using resources from these sessions in discussions with colleagues or to train others. In particular, challenges posed by vaccination hesitancy and program implementation were cited most frequently as the content they would share with field staff. In addition, 52 of the 228 respondents specifically mentioned the knowledge that they gained, receiving the most up-to-date and accurate information, learning ways to communicate about the vaccine, the interactive learning dynamic, and hearing about others’ experiences as the most helpful components of the initiative (Table 3).

**Table 3:** Survey respondents’ use of learnings and resources from this series

Use of learnings and resources	Quote	Participant type
Timely updates	“The resources help me to follow up with the updated information of vaccination.”	Health worker
	“This webinar empowered me in making evidence-based recommendations at the national level on COVID-19 planning, deployment, rollout, monitoring and evaluation, and the overall management of COVID-19 vaccines. Without these webinars, I wouldn't have had that type of timely information when I most needed it as a regulatory focal point in my country.”	Public health
	“They contained information that was up to date and as well applicable as it was time for planning for vaccine deployment at my location so I felt my needs were being supported all the time.”	Public health
Knowledge/confidence	“I improved my knowledge as a lead nurse.”	Health worker
	“The knowledge I received is immeasurable.”	Health worker
	“My knowledge and understanding about the operational aspects of COVID-19 vaccine rollout improved.”	Other
	“This information helped me and my family be in a better position to take care of ourselves and the community around us.”	Health worker
Planning/action	“I drafted and proposed technical guidance on regulatory matters on the COVID-19 vaccine to key staff in the Ministry of Health who are involved in the overall COVID-19 task force.”	Public health
	“I could better review the country’s NDVP.”	Other
	“I used the webinar resources to counsel the community about the vaccine and resolve misleading information about the vaccine.”	Public health
Sharing/training others	“I shared some resources with colleagues particularly on vaccine hesitancy and program implementation. We had a great discussion on how we can not only implement our program better but a possibility to find opportunities in the COVID-19 vaccine supply in African countries.”	Health worker
	“The knowledge gained was disseminated to field staff through meetings in local languages.”	Health worker
	“I used attached documents after each session to read them and discuss with colleagues to develop and instruct based on our local context.”	Public health

**Fig. 2:**
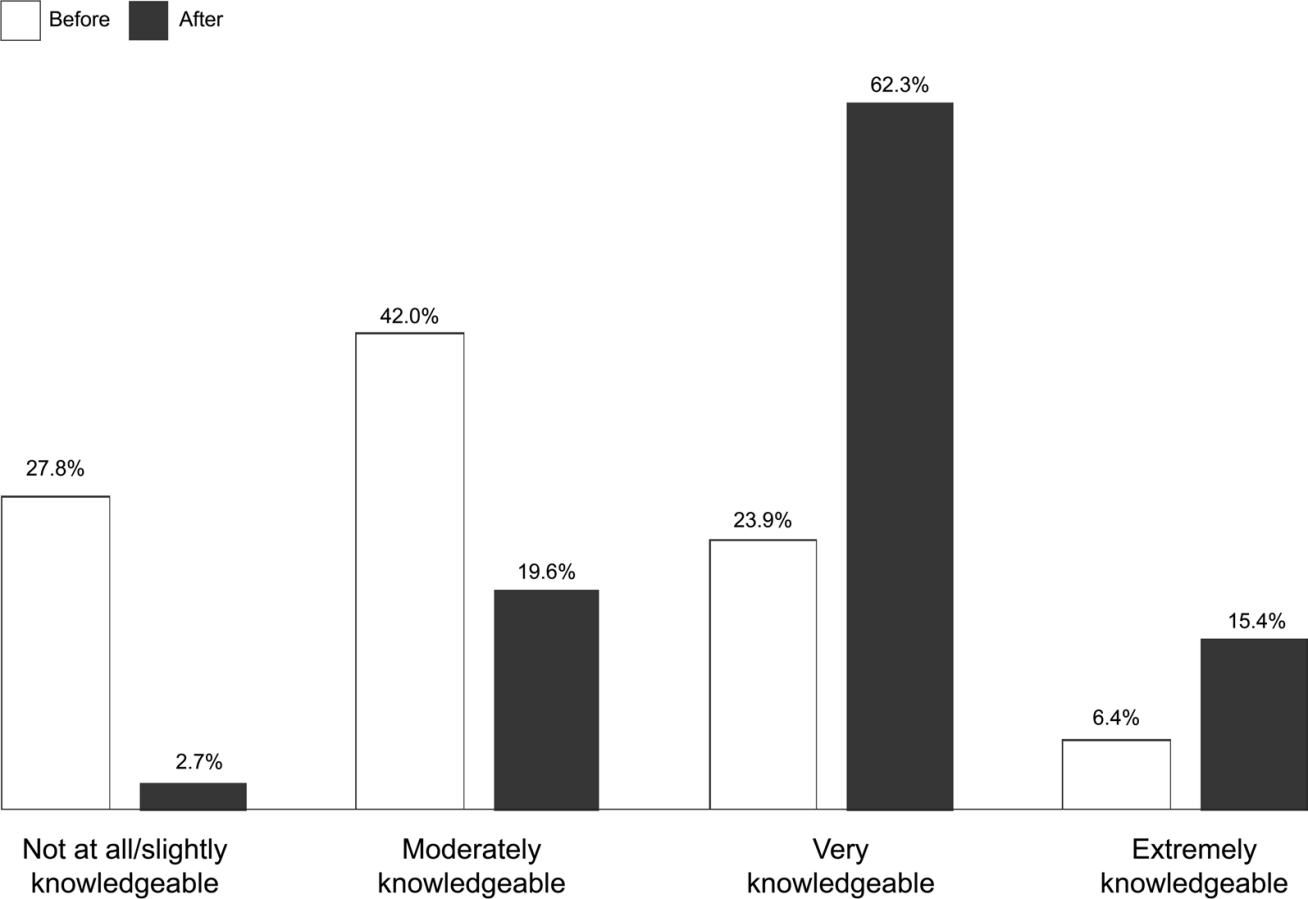
Self-reported knowledge of session topic (post-session survey respondents, *n* = 3062)

In post-session surveys, respondents rated their post-session knowledge significantly higher than before the session (Fig. 2) (*P* < 0.001). In the post-series survey, most respondents (79.6%) reported that their confidence in preparing for and rolling out COVID-19 vaccination increased “a lot” because of the initiative. However, frontline providers were less likely to report an increase in confidence than public health practitioners or other occupations (72.5% vs. 86.4% vs. 85.7%, *P* = 0.04). Additionally, participants who attended four or more sessions were more likely to report their confidence increased “a lot” compared to those who attended fewer sessions (83.8% vs. 65.4%, *P* = 0.004).

In the post-session surveys, 70% of participants reported that they will “definitely use” the knowledge gained from this series in their work; public health professionals were most likely to report this (Table 2). An additional 19.6% will “probably use” and 8.6% would “possibly use” this knowledge in their work. A number of respondents highlighted the value of their knowledge gains related to COVID-19 vaccines in their responses to the open-ended post-series survey, including understanding how to roll-out the vaccines and how to talk to friends and their communities about the vaccine (Table 3).

There is also evidence that the webinar series led to planning and action. Within the post-series survey, 19 participants responded to the open-ended question that this series empowered them to take action and advocate for changes or policies; helped them to make evidence-based recommendations related to COVID-19 vaccination planning, deployment, monitoring and evaluation, and the overall management of COVID-19 vaccine programs; provided information and resources participants used to draft and propose technical guidance on COVID-19 vaccine regulatory matters; and helped to resolve misleading information about COVID-19 vaccine delivery. Quotes from participants are available in Table 3.

Respondents provided feedback on how to improve for the future. Of the 228 respondents to the post-series survey, 17 suggested engaging more types of stakeholders (nationally, regionally, and globally), particularly government officials, who may be involved in COVID-19 vaccination planning and deployment. In addition, 15 respondents recommended adding more tangible resources (e.g., handouts and resources to teach others) and practical examples specific to LMIC contexts.

## Discussion

The *COVID-19 Vaccination: Building Global Capacity* initiative created a model of dynamic, interactive learning at scale. This initiative reflected WHO’s desire to rapidly disseminate guidance, tools, and resources for effective deployment and administration of COVID-19 vaccines. WHO and its partners also used this platform to answer questions and promote peer learning through inclusion of country case studies. Participants used this initiative to ask technical experts questions and share comments, familiarize themselves with new guidance and insights, flag challenges related to COVID-19 vaccination, and receive support for their COVID-19 vaccination planning and introduction.

The interactive nature of this digital learning initiative allowed WHO to hear COVID-19 vaccination stakeholder experiences in real time as they prepared and implemented COVID-19 vaccination delivery programs. The questions and comments during the sessions, in the post-session feedback surveys, and the discussion fora served as real-time feedback mechanisms that the CRD workstream used to identify needs and develop new resources accordingly. In particular, the mobile app enabled robust dialogue with participants. Resources were developed based on questions or requests from this initiative, including job aids explaining why there may be extra doses in a vaccine vial, how to communicate to pregnant women about COVID-19 vaccination, and how to manage vaccines without vaccine vial monitors at vaccination service points.

Although the feedback received from this initiative was overwhelmingly positive, a key limitation is the small percentage of participants who completed the post-series survey. While the participants who completed the post-series survey were more likely to have participated in more sessions, the small sample size (228 or 7.5%) impedes our ability to generalize our findings.

Traditionally, WHO headquarters receives feedback on the guidance and education resources it produces primarily from WHO regional and national staff and/or from ministries of health. The structure and format of the *COVID-19 Vaccination: Building Global Capacity* initiative allowed a wider range of stakeholders (including health workers, ministry of health program staff, students, and the public) to engage directly with technical experts, ask questions, and share their experiences. The results of the evaluation of this initiative highlight the strong interest of local, national, regional, and global stakeholders in receiving real-time resources through an interactive capacity-building program and having communication channels through which they can ask questions related to COVID-19 vaccination and receive answers from global experts. As such, the *COVID-19 Vaccination: Building Global Capacity* initiative allowed WHO to support the development of potential champions for COVID-19 vaccination globally. The modular nature of the program allowed participants to attend sessions that were relevant to their work and provided an online curated learning resource that could be used as a participant’s role changed or as their country’s COVID-19 immunization program evolved.

Additionally, the initiative provided a trusted source of information during a period where misinformation constituted a significant global challenge [24–26]. As participants highlighted through the post-session and post-series surveys, the knowledge gained from the webinars helped them to spread accurate information and encourage others to learn about COVID-19 vaccines. Ensuring access to real-time and accurate information on COVID-19 vaccines and vaccination increased confidence in preparing for and rolling out COVID-19 vaccination, particularly for public health officers at the national and subnational level, and helped participants to respond to the global COVID-19 pandemic and infodemic.

## Conclusion

WHO, TechNet-21, Project ECHO, and other partners are now considering how best to incorporate learnings from this initiative into future digital health capacity-building efforts for emergency response and routine health programs. The findings align with previous studies suggesting that interactive needs- and people-centered digital health can be effectively used for capacity building in LMICs [12, 14]. For example, participants from the African region comprised about 46% of the total attendees of this series.

WHO and TechNet-21 are considering innovative tools and approaches that can be used to enhance their capacity-building programs. The overwhelmingly positive feedback from this initiative and participants’ requests for more inclusive digital learning opportunities demonstrate that there is strong interest in further using digital capacity-building programs to promote both rapid dissemination of normative guidance and peer learning. As such, WHO and TechNet-21 are exploring opportunities to adapt or develop current learning initiatives to enable learning and community engagement by leveraging low-bandwidth, low-cost apps and videoconferencing technology. Using these platforms offers an opportunity for WHO and its partners to reach and interact with stakeholders at scale. In light of the *COVID-19 Vaccination: Building Global Capacity* experience, WHO technical experts have expressed interest in conducting similar types of interactive initiatives for other pathogen prevention and control programs, such as seasonal influenza and antimicrobial resistance.

## Data Availability

All *COVID-19 Vaccination: Building Global Capacity* session materials are publicly available on TechNet-21 [3]. The datasets analyzed during the current study are available from the corresponding author on reasonable request.

## Annex 1. COVID-19 Vaccination: Building Global Capacity sessions

**Table:**

Session number	Session title	Session description	Target audience	Date (2021)
1	National deployment and vaccination plan (NDVP) and allocation processes	Provided national and subnational focal points in countries with an overview of the NDVP preparation, submission, and review process	Public health officials	9 February
2	Interpersonal communications for COVID-19 vaccines	Focused on interpersonal communications techniques and key messages before, during, and after COVID-19 vaccination sessions	Health workers	16 February
3	Indemnification and liability	Provided key information and updates on COVID-19 vaccine indemnification and liability through the COVAX Facility. It explained the process for countries to prepare for indemnification and liability for COVID-19 vaccines, described the process for countries to access the No Fault Compensation Program, and outlined how countries can access support as they develop their national legislation	Public health officials	23 February
4	Infection prevention and control for COVID-19 vaccine introduction	Focused on the core infection prevention and control principles for COVID-19 vaccine introduction	Health workers	2 March
5	Regulatory and procurement	Provided key information on regulatory and procurement aspects for COVID-19 vaccines	Public health officials	9 March
6	Understanding COVID-19 vaccines—safety and efficacy	Presented how COVID-19 vaccines work and focused on responding to participants’ questions related to safety and efficacy	Health workers	16 March
7	Generating acceptance and demand for COVID-19 vaccines	Highlighted the package of resources available to support increased understanding of national enablers and barriers for COVID-19 vaccines	Public health officials	23 March
8	Reporting on COVID-19 vaccines (monitoring and Adverse Events Following Immunization)	Provided health workers with an overview of why reporting is important and what happens to the data after they are submitted	Health workers	30 March
9	Vaccination strategies for COVID-19 vaccination	Provided an overview of the Strategic Advisory Group of Experts on Immunization priority road map and considerations for how to reach vulnerable populations	Public health officials	6 April
10	Supply and logistics	Highlighted the COVID-19 vaccine supply and logistics guidance and provided key information about the unique requirements for the different vaccines	Health workers	13 April
11	Mobilizing financing resources for scale-up of COVID-19 vaccination	Provided an overview of the process for mobilizing financial resources and WHO’s partner platform as a mechanism to highlight resource needs	Public health officials	20 April
12	Microplanning and supportive supervision for COVID-19 vaccination	Explained the key components of microplanning for COVID-19 vaccination and promoted use of supportive supervision techniques to improve staff performance	Public health officials	4 May
13	Private sector engagement	Provided examples of how private sector partners can support COVID-19 vaccination	Public health officials	18 May
14	Monitoring and evaluating COVID-19 vaccine introduction: intra-action review and COVID-19 vaccine post-evaluation	Introduced key aspects of the monitoring and evaluation guidance and the COVID-19 post-evaluation process, which includes mini COVID-19 post-introduction evaluations (mini cPIEs) and full cPIEs	Public health officials	1 June
15	Adaptive leadership for COVID-19 vaccine introduction	Concluded the webinar series with a call to action for stakeholders to consider how they can take advantage of opportunities to strengthen COVID-19 vaccine introduction	Public health officials	15 June

## Abbreviations

ACT: Access to COVID-19 tools
CRD: Country readiness and delivery
LMIC: Low- and middle-income country
MOOCs: Massive open online courses
UNICEF: United Nations Children’s Fund
WHO: World Health Organization
